# Effects of Porosity and Mixed Convection on MHD Two Phase Fluid Flow in an Inclined Channel

**DOI:** 10.1371/journal.pone.0119913

**Published:** 2015-03-24

**Authors:** Jafar Hasnain, Zaheer Abbas, Muhammad Sajid

**Affiliations:** 1 Department of Mathematics, The Islamia University of Bahawalpur, Bahawalpur, Pakistan; 2 Theoretical Physics Division, PINSTECH, P.O. Nilore, Islamabad, Pakistan; Bascom Palmer Eye Institute, University of Miami School of Medicine;, UNITED STATES

## Abstract

The present study deals with the flow and heat transfer analysis of two immiscible fluids in an inclined channel embedded in a porous medium. The channel is divided in two phases such that a third grade fluid occupies the phase I and a viscous fluid occupies the phase II. Both viscous and third grade fluids are electrically conducting. A constant magnetic field is imposed perpendicular to the channel walls. The mathematical model is developed by using Darcy's and modified Darcy's laws for viscous and third grade fluids respectively. The transformed ordinary differential equations are solved numerically using a shooting method. The obtained results are presented graphically and influence of emerging parameters is discussed in detail.

## Introduction

Fluid flow nested with porous medium is a common phenomena in nature such as transport of water in living plants, trees and fertilizers or wastes in soil. In the industry, the flow through porous medium has acquired much attention in the view of its promising applications in several technological processes for example, filtration, catalysis, chromatography, petroleum exploration and recovery, cooling of electronic equipment.

The practical applications of the non-Newtonian fluids in engineering and industry have captivated a number of investigators during the past few years. Some of the important applications are aerodynamic heating, electrostatic precipitation, petroleum industry, synthetic fibres, manufacture of intumescent paints for fire safety applications and paper production. Several models have been proposed to describe the physical behaviour and properties of non-Newtonian fluids. These models exhibit a non linear relationship between the stress and rate of strain. Amongst them, the fluid of third grade, which form a subclass of the fluids of differential type [1], is considered in the present study. This model is known to acquire the non-standard features such as normal stress effects, shear thinning or shear thickening. The third grade fluid model was thermodynamically studied in depth by Fosdick and Rajagopal [2]. In another study [3], Rajagopal showed that the third grade fluids exhibit different features as compared to the Newtonian and second grade fluids. The steady, laminar flow of a third grade fluid through a porous flat channel was studied by Ariel [4]. Sajid et al. [5] investigated the steady flow of third grade fluid past a horizontal porous flat plate with partial slip. They solved the arising non-linear problem numerically using a finite element method. The flow of the non-Newtonian fluids in the vicinity of porous medium has vital applications in enhanced oil recovery, insulation systems, filtration processes and composite manufacturing processes, etc. One can refer to some of the studies [6–10] in this regard.

From the industrial and engineering point of view, the study of two-phase flow is of considerable practical interest. Counter current liquid-liquid flow is an example of two phase flow in an inclined channel and has applications in process industry. Ullmann et al. [11] investigated the counter current flow in a vertical, off vertical and inclined columns. Another example of the flow of two phase fluid down an inclined plane is the manufacturing of photographic films. For details the readers are referred to the book by Chandrasekhar [12]. The two phase fluid is also important in studying the blood flow particularly for the Fahraeus-Lindqvist effect. Majhi and Usha [13] investigated the F-L effect by considering blood as a third grade non-Newtonian fluid. The two phase flow also occurs in many industrial processes such as steam generators and condensers, refrigeration, liquid sprays, food manufacturing and medical applications. Furthermore, the study of the flow and heat transfer phenomena of two immiscible fluids through pipelines and wells has advanced rapidly in recent decades because of its wide applications in petroleum industry. The researchers’ attention towards these types of the problem originates from the possibility of reducing the power required to pump oil in a pipeline by suitable addition of water. A wealth of literature can be found on a stratified laminar flow of two immiscible fluids with different geometries. Packham and Shail [14] studied the laminar flow of two immiscible fluids through a horizontal pipe. MHD two-phase fluid flow in a rectangular channel was investigated by Shail [15]. Lohrasbi and Sahai [16] considered MHD two-phase flow and heat transfer in a horizontal parallel-plate channel and reported analytical solutions for the velocity and temperature profiles for the case where only one of the fluids is electrically conducting. The perturbation analysis for the problem of two-phase magnetohydrodynamic flow and heat transfer in a horizontal channel was investigated by Malashetty and Leela [17]. In another study, Malashetty and Leela [18] examined the two-phase flow and heat transfer situation in a horizontal channel for which both phases are electrically conducting and presented the closed form solutions. Malashetty and Umavathi [19] investigated the two-phase MHD flow and heat transfer in an inclined channel. The problem of fully developed free convection two fluid magnetohydrodynamic flow in an inclined channel was presented by Malashetty et al. [20]. Umavathi et al. [21] studied the problem of unsteady oscillatory flow and heat transfer of two viscous immiscible fluids through a horizontal channel with isothermal permeable walls. Kumar et al. [22] discussed the mixed convective flow and heat transfer in a vertical channel for the situation where only one of the phases is electrically conducting. Recently Abbas et al. [23] investigated the velocity and thermal slip effects in MHD flow and heat transfer of two-phase viscous fluid.

All the studies in the above paragraph pertain to the study of two immiscible fluids in a channel in the absence of porous medium. However, the flow problem through a porous medium channel finds many applications in geothermal and geophysical applications. These applications include underground disposal of nuclear wastes, spreading of chemical pollutants in water-saturated soil, migration of moisture in fibrous insulation, structure reaction and gas assisted injection molding, die filling processes, clean-up of refineries and extraction of geothermal energy. Nakayama et al. [24] made an analysis on forced convection in a channel filled with a Brinkman-Darcy porous medium and presented both exact and approximate solutions. Vafai and Kim [25] have studied and reported an exact solution for forced convection in a channel filled with a porous medium. The flow and heat transfer characteristics of Oberbeck convection of a couple stress fluid in a vertical porous stratum was investigated by Umavathi and Malashetty [26]. Chamkha [27] performed an analysis on the flow of two-immiscible fluids in porous and non-porous channel. Umavathi et al. [28] examined the unsteady oscillatory flow and heat transfer in a horizontal composite porous medium in the presence of viscous dissipation. In a research article, Singh et al. [29] addressed the transient as well as non-Darcian effects of laminar natural convection flow in a vertical channel partially filled with porous medium. Chuhan and Agrawal [30] analyzed the fully developed MHD mixed convection flow in a vertical channel partially filled with clear fluid and partially filled with a fluid-saturated porous medium. Recently, Sivaraj et al. [31] investigated the MHD mixed convective flow of viscoelastic and viscous fluids in a vertical porous channel and obtained the exact solution.

The aim of the present investigation is to study the problem of laminar flow of incompressible and electrically conducting immiscible fluids in an inclined channel through a porous medium. In the present study we have analyzed a two-phase flow down an inclined plane by considering one of the fluid as a third grade non-Newtonian fluid. The third grade fluid exhibits the shear thinning characteristics, therefore the proposed results modify the previous analysis when both the fluids are taken to be Newtonian. The channel is filled with two immiscible fluids namely viscous fluid and the third grade fluid. A transverse magnetic field is applied perpendicular to the flow of the fluid. Section 2 contains the details of the developed mathematical model. The detail of the numerical method used is presented in section 3. Numerical results and their discussion are presented in section 4. Section 5 contains some concluding remarks.

## Formulation of the Problem

**Fig 1:**
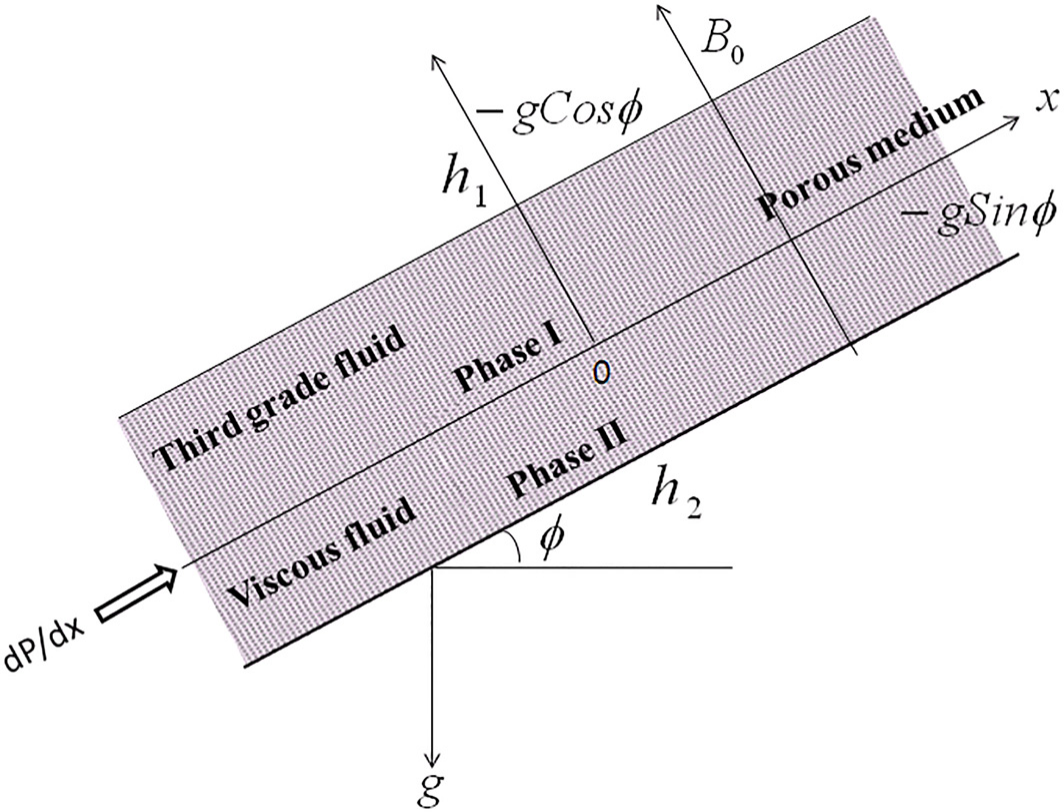
Physical configuration and coordinate system.

We consider a steady, incompressible, laminar and fully developed flow between two infinite parallel plates extending in *x*–and *z*–direction making an inclination *ϕ* with the horizontal. The geometry of the problem is presented in Fig. 1. The regions 0 ≤*Y ≥h*_*1*_ and—*h*_2_≤*Y ≥*0 are denoted as phase I and phase II, respectively. The phase I is filled with an electrically conducting third grade fluid of density *p*_1_, viscosity *μ*_1_, electrical conductivity σ_1_ and thermal conductivity *K*_1_ where as the phase II is occupied with an electrically conducting viscous fluid of density *p*_2_, viscosity *μ*_2_, electrical conductivity σ_2_ and thermal conductivity *K*_*2*_. A uniform magnetic field of strength ***B*** is applied perpendicular to the flow field and the flow is saturated in porous medium. The fluid properties of both the fluids are taken as constant and the flow of both phases is driven by constant pressure gradient (-∂*p /*∂*x*). The walls are maintained at constant temperature *T*_*w1*_ and *T*_*w2*_ at *y = h*_*1*_ and *y = h*_*1*_ respectively. Under these assumptions, the governing equations for the flow phenomena in the vector form are given as

$$\nabla \cdot V_{i}=0,$$ (1)

$$\rho_{i}\frac{dV_{i}}{dt}=\text{div}\tau_{i}+J\times B+R_{i}+\rho_{i}g\beta_{i}\sin\phi \left(T_{i}-T_{w_{2}}\right),$$ (2)

ρicpdTidt=τi⋅Li+Ki∇2Ti. (3)

where *i* = 1,2 respectively describe the phases I and II, *d*.*/ dt* is the material derivative, ***J*** is the electric current density, ***R***_*i*_ are the Darcy’s resistances, ***L***_*i*_ are the velocity gradients, *c*_*p*_ is the specific heat, ***g*** is acceleration due to gravity, *p*_*i*_ are densities, *β*_*i*_ are the coefficient of thermal expansions, *T*_*i*_ are the temperatures, *K*_*i*_ re the thermal conductivities, **τ**_1_ and **τ**_2_ are the Cauchy stress tensors for third grade fluid and viscous fluid respectively defined as

$$\tau_{1}=-pI+\mu_{1}A_{1}+\alpha_{1}A_{2}+\alpha_{2}A_{1}{}^{2}+\beta_{3}(\text{tr}A_{1}{}^{2})A_{1},$$ (4)

$$\tau_{2}=-pI+\mu_{2}A_{1},$$ (5)

where *p* is pressure, ***I*** is identity tensor, *α*_*1*,_ *α*_*2*_ and *β*_3_ are the material constants. The Rivlin Ericksen tensors are defined as

$$A_{1}=\nabla V+{\left(\nabla V\right)}^{\text{T}},$$ (6)

and

$$A_{2}=\frac{dA_{1}}{dt}+A_{1}\nabla V+{\left(\nabla V\right)}^{\text{T}}A_{1}.$$ (7)

The value of the Darcy’s resistance for third grade fluid is given by

$$R_{1}=-\left[\mu_{1}+2\beta_{3}{\left(\frac{du_{1}}{dy}\right)}^{2}\right]\frac{\varphi V_{1}}{k^{*}},$$ (8)

and for viscous fluid

$$R_{2}=-\frac{\mu_{2}\varphi }{k^{*}}V_{2},$$ (9)

where *k* ^*^ and *φ* are the permeability and porosity of the porous medium respectively.

For the present problem the velocity field is given by

$$V_{i}=\left(u_{i}\left(y\right),0,0\right),$$ (10)

where *u*_*i*_ (*I* = 1,2) are the *x*- component of the velocities.

Using Equations (4)–(10) in Equations (2)–(3), we get the following equations

For Phase I

$$\mu_{1}\frac{d^{2}u_{1}}{dy^{2}}+\rho_{1}g\beta_{1}\sin\phi \left(T_{1}-T_{w_{2}}\right)-\sigma_{1}B_{0}^{2}u_{1}+6\beta_{3}\frac{d^{2}u_{1}}{dy^{2}}{\left(\frac{du_{1}}{dy}\right)}^{2}-\frac{\varphi }{k^{*}}\left(\mu_{1}+2\beta_{3}{\left(\frac{du_{1}}{dy}\right)}^{2}\right)\;u_{1}=\frac{\partial p}{\partial x},\;$$ (11)

$$K_{1}\frac{d^{2}T_{1}}{dy^{2}}+\mu_{1}{\left(\frac{du_{1}}{dy}\right)}^{2}+2\beta_{3}{\left(\frac{du_{1}}{dy}\right)}^{4}+\sigma_{1}B_{0}^{2}u_{1}^{2}=0.$$ (12)

For Phase II

$$\mu_{2}\frac{d^{2}u_{2}}{dy^{2}}+\rho_{2}g\beta_{2}\sin\phi \left(T_{2}-T_{w_{2}}\right)+\sigma_{2}B_{0}^{2}u_{2}-\frac{\varphi \;\mu_{2}}{k^{*}}u_{2}=\frac{\partial p}{\partial x},\;$$ (13)

$$K_{1}\frac{d^{2}T_{2}}{dy^{2}}+\mu_{2}{\left(\frac{du_{2}}{dy}\right)}^{2}+\sigma_{2}B_{0}^{2}u_{2}^{2}=0.$$ (14)

With reference to the Fig. 1, the boundary and interface conditions on velocity and temperature are

$$\begin{array}{c} u_{1}\left(h_{1}\right)=0,\;\\ u_{1}\left(0\right)=u_{2}\left(0\right),\;\\ u_{2}\left(h_{2}\right)=0,\;\\ \mu_{1}\frac{du_{1}}{dy}+2\beta_{3}{\left(\frac{du_{1}}{dy}\right)}^{3}=\mu_{2}\frac{du_{2}}{dy}\text{at}y=0,\; \end{array}$$ (15)

$$\begin{array}{c} T_{1}\left(h_{1}\right)=T_{w_{1}},\;\\ T_{1}\left(0\right)=T_{2}\left(0\right),\;\\ T_{2}\left(-h_{2}\right)=T_{w_{2}},\;\\ K_{1}\frac{dT_{1}}{dy}=K_{2}\frac{dT_{2}}{dy}\text{at}y=0. \end{array}$$ (16)

Incorporating the following dimensionless variables and parameters

$$\begin{array}{c} u_{i}^{*}=u_{i}/{\bar{u}}_{1}\text{,}y_{i}^{*}=y_{i}/h_{i},\;\theta =\left(T-T_{w_{2}}\right)/\Delta T,\;\\ m=\mu_{1}/\mu_{2},\;k=K_{1}/K_{2},\;h=h_{2}/h_{1},\;n=\rho_{2}/\rho_{1},\;b=\beta_{2}/\beta_{1},\;s=\sigma_{2}/\sigma_{1},\;\\ Gr=g\beta_{1}h_{1}^{3}\Delta T/\nu_{1}^{2},\;M=B_{0}h_{1}\sqrt{\sigma_{1}/\mu_{1}},\;\mathrm{Pr}=\mu_{1}C_{p}/K_{1},\;\beta =\beta_{3}{\bar{u}}_{1}^{2}/h_{1}^{2},\;\lambda =h_{1}^{2}\varphi /k^{*},\;\\ P=\left(h_{1}^{2}/\mu_{1}{\bar{u}}_{1}\right)\;\left(\partial p/\partial x\right),\;Re={\bar{u}}_{1}h_{1}/\nu_{1},\;Ec={\bar{u}}_{1}^{2}/C_{p}\Delta T. \end{array}$$ (17)

Here *Gr* is the Grashof number, *Ec* is the Eckert number, Pr is the Prandtl number, Re is the Reynolds number, *M* is the Hartmann number, *β* is the third grade parameter, λ is the porosity parameter, *P* is the nondimensional pressure gradient and ${\bar{u}}_{1}$ is the average velocity.

With the above nondimensional quantities, our problem takes the following form

Phase I

$$\frac{d^{2}u_{1}}{dy^{2}}+\frac{Gr\sin\phi }{Re}\theta_{1}-M^{2}u_{1}+6\beta {\left(\frac{du_{1}}{dy}\right)}^{2}\frac{d^{2}u_{1}}{dy^{2}}-\lambda \left(1-2\beta \frac{d^{2}u_{1}}{dy^{2}}\right)\;u_{1}=P,\;$$ (18)

$$\frac{d^{2}\theta_{1}}{dy^{2}}+\varepsilon {\left(\frac{du_{1}}{dy}\right)}^{2}+2\beta \varepsilon {\left(\frac{du_{1}}{dy}\right)}^{4}+\varepsilon M^{2}u_{1}^{2}=0.$$ (19)

Phase II

$$\frac{d^{2}u_{2}}{dy^{2}}+\frac{Gr}{Re}bmnh^{2}\sin\phi \theta_{2}-msh^{2}M^{2}u_{2}-h^{2}\lambda u_{2}=mh^{2}P,\;$$ (20)

$$\frac{d^{2}\theta_{2}}{dy^{2}}+\varepsilon \left(\frac{k}{m}\right)\;{\left(\frac{du_{2}}{dy}\right)}^{2}+M^{2}\varepsilon kh^{2}su_{2}^{2}=0.$$ (21)

The asterisks have been dropped for simplicity and ε <<1 (= Pr *Ec*) because in most of the practical problems the Eckert number is very small and is of order 10^−5^ (see [20]).

The velocity, temperature and interface boundary conditions (15) and (16) in nondimensional form are

$$\begin{array}{c} u_{1}\left(1\right)=0,\;\\ u_{1}\left(0\right)=u_{2}\left(0\right),\;\\ u_{2}\left(-1\right)=0,\;\\ \frac{du_{1}}{dy}+2\beta {\left(\frac{du_{1}}{dy}\right)}^{3}=\left(1/mh\right)\;\frac{du_{2}}{dy}\text{ at }y=0. \end{array}$$ (22)

$$\begin{array}{c} \theta_{1}\left(1\right)=1,\;\\ \theta_{1}\left(0\right)=\theta_{2}\left(0\right),\;\\ \theta_{2}\left(-1\right)=0,\;\\ \frac{d\theta_{1}}{dy}=\left(1/kh\right)\;\frac{d\theta_{2}}{dy}\text{at}y=0. \end{array}$$ (23)

## Numerical Solution

The solution of the problem in terms of velocity and temperature distributions is obtained numerically by employing a shooting method with Runge-Kutta algorithm for various values of the parameters for example: *β*,*λ*,*m*,*s*,*h* and *φ* In order to solve the Equations (18)–(23) by shooting method we need to convert the boundary value problems into initial value problems by assuming

$$u_{1}{}^{\prime }\left(1\right)=p_{1},\;\theta_{1}{}^{\prime }\left(1\right)=p_{2},\;u_{2}{}^{\prime }\left(-1\right)=s_{1},\;\theta_{2}{}^{\prime }\left(-1\right)=s_{2}.$$ (24)

Differentiating (Equation 18) w. r. t *p*_1,_ (Equation 19) w. r. t *p*_2,_ (Equation 20) w. r. t *s*_1,_ (Equation 21) w. r. t *s*_*2*_

$$\frac{d^{2}U_{1}}{dy^{2}}-M^{2}U_{1}+6\beta {\left(\frac{du_{1}}{dy}\right)}^{2}\frac{d^{2}U_{1}}{dy^{2}}+12\beta \frac{du_{1}}{dy}\frac{dU_{1}}{dy}\frac{d^{2}u_{1}}{dy^{2}}-\lambda \left(1-2\beta \frac{d^{2}u_{1}}{dy^{2}}\right)\;U_{1}+2\lambda \beta \frac{d^{2}U_{1}}{dy^{2}}u_{1}=0,\;$$ (25)

$$\frac{d^{2}\vartheta_{1}}{dy^{2}}=0,$$ (26)

$$\frac{d^{2}U_{2}}{dy^{2}}-msh^{2}M^{2}U_{2}-h^{2}\lambda U_{2}=0,\;$$ (27)

$$\frac{d^{2}\vartheta_{2}}{dy^{2}}=0.$$ (28)

subject to initial conditions

$$U_{1}\left(1\right)=0,\;U_{1}{}^{\prime }\left(1\right)=1,$$ (29)

$$\vartheta_{1}\left(1\right)=0,\;\vartheta_{1}{}^{\prime }\left(1\right)=1,$$ (30)

$$U_{2}\left(-1\right)=0,\;U_{2}{}^{\prime }\left(-1\right)=1,$$ (31)

$$\vartheta_{2}\left(-1\right)=0,\;\vartheta_{2}{}^{\prime }\left(-1\right)=1,$$ (32)

where U_1_ represents derivative with respect to *p*_1_,ϑ_1_ represents derivative with respect to *p*_2_,*U*_2_ represents derivative with respect to *s*_1_, ϑ_2_ represents derivative with respect to *s*_*2*_

Our numerical procedure works in the following way. We choose a suitable value for parameters

*P*_1_, *P*_2_, *s*_*1*_ and *s*_2_ then R-K method is applied to integrate the initial value problems (18)-(21), (25), (26), (27) and (28) with the initial conditions (22)-(23), (29), (30), (31) and (32) respectively. The values of parameters *P*_1_, *P*_2_, *s*_*1*_ and *s*_2_ are modified by using a suitable zero finding algorithm. In the present case we have used Newton’s method so that

$$p_{1}\left(\kappa +1\right)=p_{1}\left(\kappa \right)-\frac{\frac{du_{1}\left(0\right)}{dy}+2\beta {\left(\frac{du_{1}\left(0\right)}{dy}\right)}^{3}-\left(1/mh\right)\;\frac{du_{2}\left(0\right)}{dy}}{\frac{dU_{1}\left(0\right)}{dy}+2\beta {\left(\frac{dU_{1}\left(0\right)}{dy}\right)}^{3}},$$ (33)

$$p_{2}\left(\kappa +1\right)=p_{2}\left(\kappa \right)-\frac{\frac{d\theta_{1}\left(0\right)}{dy}-\left(1/kh\right)\;\frac{d\theta_{2}\left(0\right)}{dy}}{\frac{d\vartheta_{1}\left(0\right)}{dy}},$$ (34)

$$s_{1}\left(\kappa +1\right)=s_{1}\left(\kappa \right)-\frac{u_{1}\left(0\right)-u_{2}\left(0\right)}{U_{2}\left(0\right)},$$ (35)

$$s_{2}\left(\kappa +1\right)=s_{2}\left(\kappa \right)-\frac{\theta_{1}\left(0\right)-\theta_{2}\left(0\right)}{\vartheta_{2}\left(0\right)}.$$ (36)

The above process is repeated until an accuracy of 10^–6^ is achieved.

## Results and Discussion

**Fig 2:**
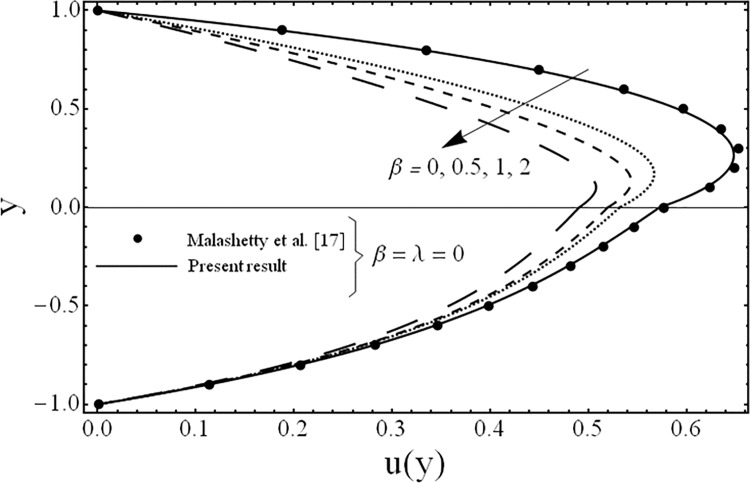
Variation of velocity profiles *u*(*y*) for several values of third grade parameter *β*.

**Fig 3:**
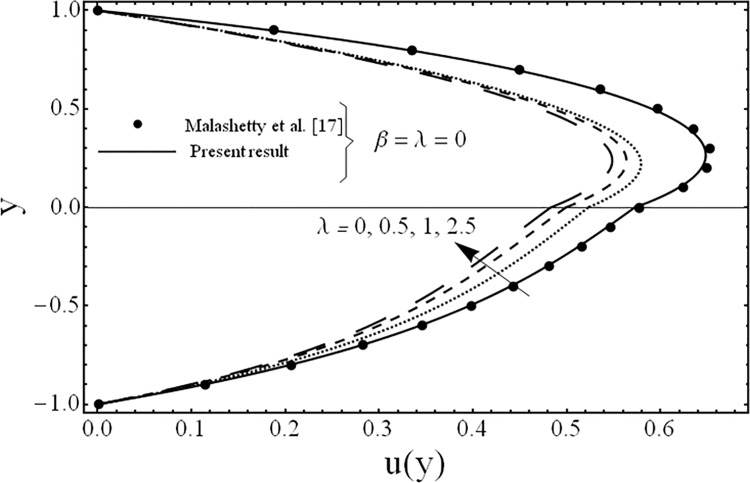
Variation of velocity profiles *u*(*y*) for several values of porosity parameter *λ*.

**Fig 4:**
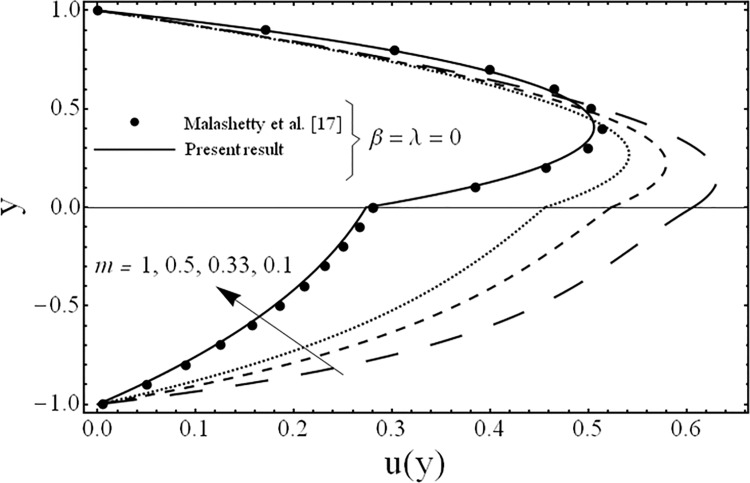
Variation of velocity profiles *u*(*y*) for several values of ratio of viscosities *m*.

**Fig 5:**
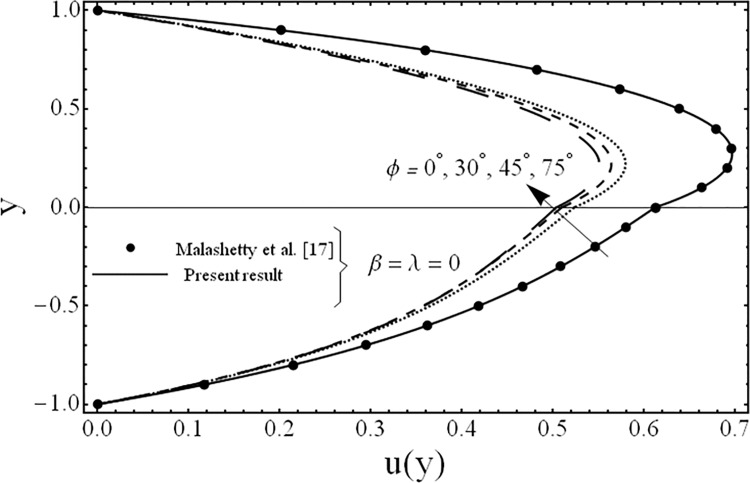
Variation of velocity profiles *u*(*y*) for several values of angle of inclination *ϕ*.

**Fig 6:**
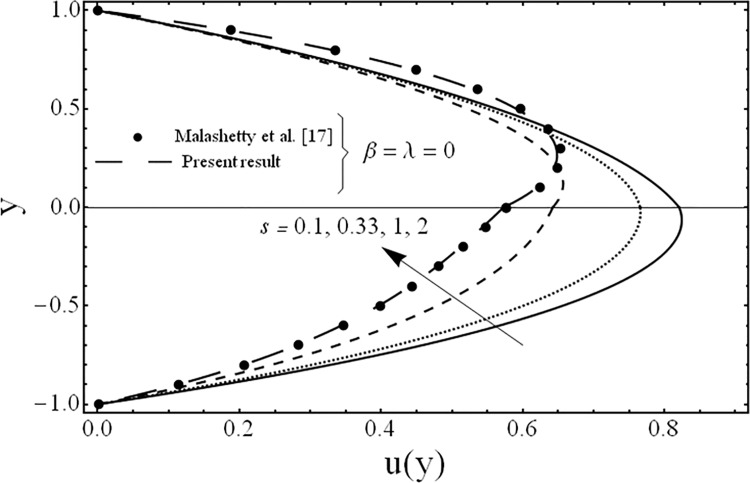
Variation of velocity profiles *u*(*y*) for several values of ratio of electrical conductivities *s*.

**Fig 7:**
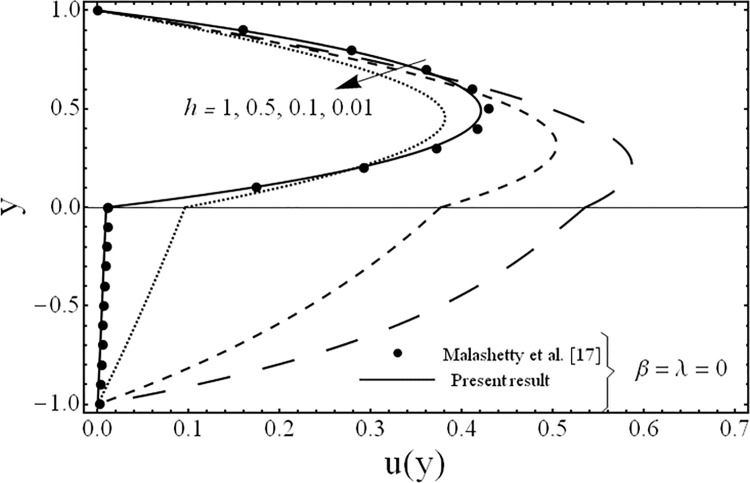
Variation of velocity profiles *u*(*y*) for several values of ratio of heights *h*.

**Fig 8:**
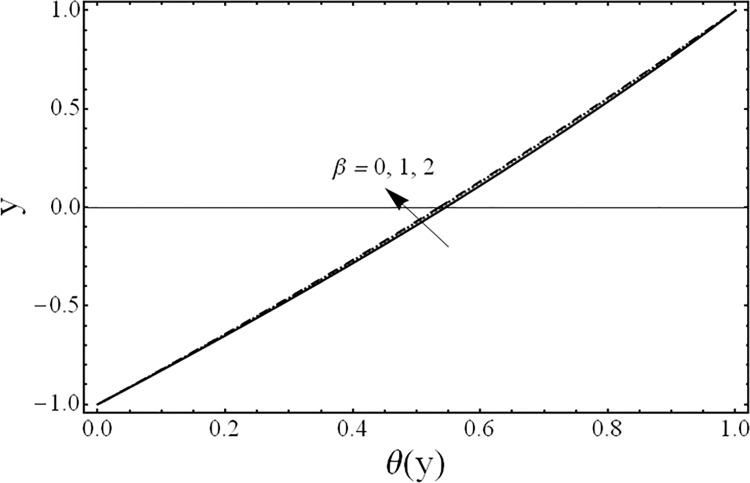
Variation of temperature profiles θ(*y*) for several values of third grade parameter *β*.

**Fig 9:**
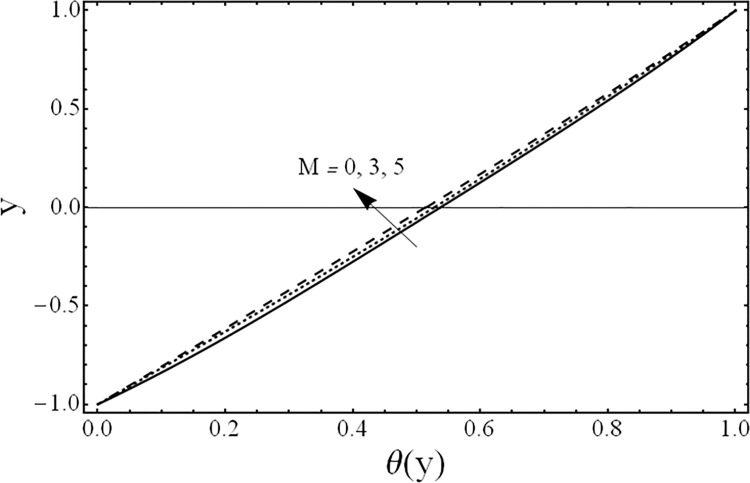
Variation of temperature profiles θ(*y*) for several values of magnetic parameter *M*.

**Fig 10:**
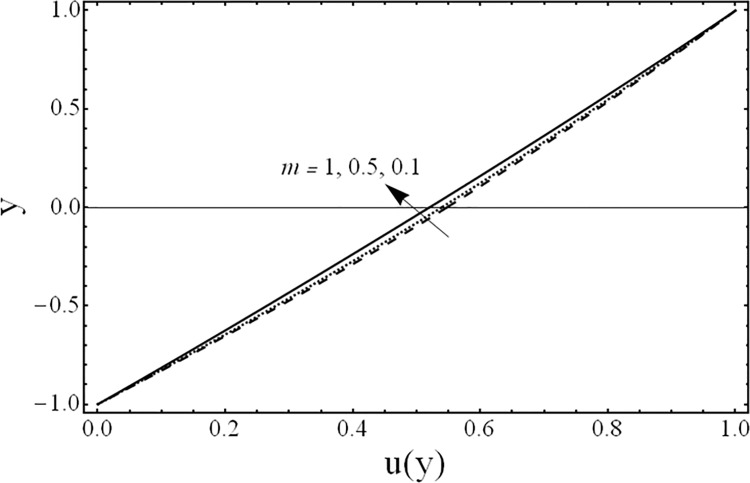
Variation of temperature profiles θ(*y*) for several values of ratio of viscosities *m*.

**Fig 11:**
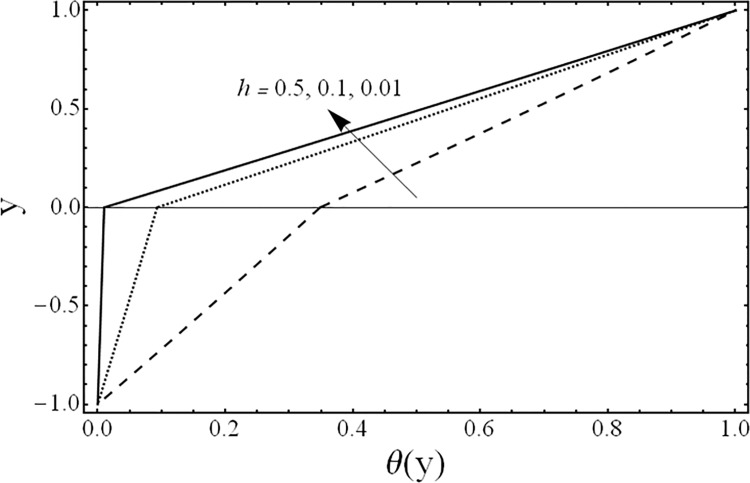
Variation of temperature profiles θ(*y*) for several values of ratio of heights *h*.

The solution of the problem in terms of velocity and temperature distributions is obtained numerically by employing a shooting method with Runge-Kutta algorithm for various values of the parameters for example the third grade parameter *β*, the porosity parameter *λ*, ratios of viscosities *m*, electrical conductivities *s*, heights of the fluids *h* and the inclination of channel *ϕ*. For physical illustrations of the numerical results Figs. 2–11 are presented and the following parameters *Gr* = 5, Re = 5, Pr = 5, *k* = 1, n = 105, *M* = 2 are fixed in this study.

The variation in the fluid velocity *u*(*y*). is shown for several values of the third grade parameter *β* in Fig. 2. From this Fig., it is clear that the fluid velocity decreases with the increasing value of the third grade parameter *β*. The cause for this change is the shear thickening effects which are increased by increasing value of the third grade parameter *β*. Moreover, it is also observed that the decrement in the fluid velocity in the upper phase is larger as compared to the fluid velocity in the lower phase due the presence of third grade fluid. Fig. 3 exhibits the effect of the porosity parameter *λ* on the fluid velocity *u*(*y*). The effect of the porosity parameter is to suppress the fluid velocity *u*(*y*). in both the phases because of the dampening effects of the Darcy's resistance. Fig. 4 depicts the effect of the ratio of the viscosities *m* on the fluid velocity *u*(*y*). From this figure, it is evident that the effects of ratio of viscosities *m* is quite interesting and the flow field is larger in the lower phase due to the smaller value of the viscosity of the fluid compared to the fluid in the upper phase. Fig. 5 presents the influence of the angle of the inclination *ϕ* on the fluid velocity *u*(*y*). It is noticed from this figure that an increase in the angle leads to a decrease in the velocity of the fluid and the flow field is larger in the case of third grade fluid. Fig. 6 is plotted to present the influence of the ratio of the electrical conductivities *s* on the fluid velocity *u*(*y*). One can see from this figure that an increase in the ratio of the electrical conductivities reduces the magnitude of fluid velocity. It is further noted that the flow field is larger in the case of third grade fluid. Fig. 7 shows the change in the fluid velocity *u*(*y*) for several values of the ratio of heights of the fluid *h* From this figure, it is evident as we increase the ratio of heights of the fluid (or channel) the magnitude of the fluid velocity decreases. It is noteworthy that in all the above figures, the present results are compared with the existing results of Malashetty et al. [20] for the case of Newtonian fluid (*β* = 0) and clear medium (*λ = 0*), and found them to be in good agreement.

Fig. 8 shows the variation in the temperature distribution *θ*(*y*) for different values of the third grade fluid parameter *β* It is noted from this figure, that an increase in third grade parameter *β* results in the reduction of fluids’ temperature. The variations in the temperature distribution *θ*(*y*) for several values of the magnetic parameter *M* are presented in Fig. 9. From this figure, it is evident that the temperature of the fluid decreases by increasing the value of the magnetic parameter *M* Furthermore, the change in the temperature distribution is more visible in the case of magnetic field as compared with third grade parameter *β*. Fig. 10 gives the influence of the ratio of the viscosities *m* on the temperature profile *θ*(*y*). It can be examined that as the ratio of viscosities increases the temperature of the fluid decreases. Moreover, the effect of the ratio of viscosities *m* on temperature *θ*(*y*) is same as that of its effect on the velocity field. Fig. 11 illustrates the effect of the ratio of the heights *h* on the temperature field *θ*(*y*). It is noticed from this figure that the magnitude of the temperature is larger in the upper phase due to its small height compared to the lower phase. From this figure, it is also evident that the effect of the ratio of heights *h* on temperature profile is same as its effect on the velocity field.

## Concluding Remarks

In this work, a study of mixed convective hydromagnetic flow of two immiscible fluids namely the third grade fluid and the viscous fluid in an inclined channel through a porous medium has been carried out. The obtained dimensionless ordinary differential equations governing the problem are solved numerically. The effects of numerous physical parameters on the fluid velocity and temperature distributions were discussed with the help of several graphs. Following conclusions have been made from this investigation which are as follows:

- The fluid velocity *u*(*y*) decreases with the increasing value of the third grade parameter *β*, the porosity parameter *λ* and the magnetic parameter *M*.
- The temperature distribution *θ*(*y*) decreases with the increasing value of the third grade parameter *β*, the porosity parameter *λ* and the magnetic parameter *M* throughout the channel.
- The velocity field in upper phase is large due to the presence of third grade fluid compared to the lower phase.
- Both the fluid velocity *u*(*y*) and temperature field *θ*(*y*) are increased as we increase the values of *h* and *m*.
